# Cell Cycle Phase Regulates Glucocorticoid Receptor Function

**DOI:** 10.1371/journal.pone.0022289

**Published:** 2011-07-29

**Authors:** Laura Matthews, James Johnson, Andrew Berry, Peter Trebble, Ann Cookson, Dave Spiller, Caroline Rivers, Michael Norman, Mike White, David Ray

**Affiliations:** 1 Developmental Biomedicine Research Group, University of Manchester, Manchester, United Kingdom; 2 Centre for Cell Imaging, University of Liverpool, Liverpool, United Kingdom; 3 Faculty of Life Sciences, University of Manchester, Manchester, United Kingdom; 4 Division of Medicine, University of Bristol, Bristol, United Kingdom; Institut de Génomique Fonctionnelle de Lyon, France

## Abstract

The glucocorticoid receptor (GR) is a member of the nuclear hormone receptor superfamily of ligand-activated transcription factors. In contrast to many other nuclear receptors, GR is thought to be exclusively cytoplasmic in quiescent cells, and only translocate to the nucleus on ligand binding. We now demonstrate significant nuclear GR in the absence of ligand, which requires nuclear localisation signal 1 (NLS1). Live cell imaging reveals dramatic GR import into the nucleus through interphase and rapid exclusion of the GR from the nucleus at the onset of mitosis, which persists into early G_1_. This suggests that the heterogeneity in GR distribution is reflective of cell cycle phase.

The impact of cell cycle–driven GR trafficking on a panel of glucocorticoid actions was profiled. In G2/M-enriched cells there was marked prolongation of glucocorticoid-induced ERK activation. This was accompanied by DNA template-specific, ligand-independent GR transactivation. Using chimeric and domain-deleted receptors we demonstrate that this transactivation effect is mediated by the AF1 transactivation domain. AF-1 harbours multiple phosphorylation sites, which are consensus sequences for kinases including CDKs, whose activity changes during the cell cycle.

In G2/M there was clear ligand independent induction of GR phosphorylation on residues 203 and 211, both of which are phosphorylated after ligand activation. Ligand-independent transactivation required induction of phospho-S211GR but not S203GR, thereby directly linking cell cycle driven GR modification with altered GR function. Cell cycle phase therefore regulates GR localisation and post-translational modification which selectively impacts GR activity. This suggests that cell cycle phase is an important determinant in the cellular response to Gc, and that mitotic index contributes to tissue Gc sensitivity.

## Introduction

Glucocorticoids (Gc) are essential for life, mediating a diverse array of effects to regulate bone and glucose homeostasis, tissue remodeling and repair, and the immune response [1]. Gc are the most potent anti-inflammatory agents known and as such, synthetic Gc are widely used in the treatment of inflammatory disease. However, a major factor limiting their clinical use is the broad variation in patient response to Gc therapy. A number of genetic factors are known to regulate Gc sensitivity, but less is known about how Gc sensitivity is regulated in-vivo [2]–[4].

Gc modulate cellular events following binding and activation of the ubiquitously expressed intracellular glucocorticoid receptor (GR) [1], [5], a member of the nuclear hormone receptor superfamily of ligand activated transcription factors [6]–[8].

In an inactive state the GR resides in the cytoplasm as part of a multi-protein complex, which includes chaperone proteins and immunophilins [5], [9]–[12]. Ligand activated GR is released from this complex and is then free to initiate non-genomic effects within the cytoplasm, and then translocate to the cell nucleus where it dimerises and binds palindromic Gc-response elements (GREs). The GR-GRE complex has the capacity to recruit either coactivator or corepressor molecules that can modify chromatin and either facilitate or inhibit transcription initiation [5], [13], [14]. However, the intracellular distribution of GR is not consistently as clearly segregated as this model would suggest, with significant nuclear GR observed even under ligand-free conditions. Other investigators have also shown that GR can move between cytoplasm and nucleus when unliganded and bound to the heat shock protein complex [15]. This aberration has also been attributed to low-level ligand activation, but other explanations have not been explored [16]–[19].

The GR contains two nuclear localization sequences, NLS1, which lies between the DNA binding domain and the ligand binding domain, and in addition NLS2, which is less-well defined, and lies within the ligand binding domain. NLS1 transports GR into the nucleus in an importin α, and importin 7 dependent manner. It now appears that import of the GR into the nucleus may also occur when GR remains bound to the heat shock protein complex, through interactions with the nuclear pore protein Nup62 [20]. In contrast to the rapid rate of ligand activated nuclear import, export of both unliganded and ligand bound GR is a slow process, taking up to 14 hours. This has recently been defined as resulting from a distinct nuclear retention domain that also lies in the hinge region between the DNA binding and ligand binding domains and which acts to oppose exportin mediated cytoplasmic relocation [21].

GR is a potent modulator of cell cycle phase, interacting with cell cycle regulating kinases and inducing arrest at the G0/G1 checkpoint [22]. Additionally, GR activity is regulated by cell cycle phase, with evidence for specific changes to transactivation function, and induction of S211GR phosphorylation; although changes to the transcriptional regulatory functions of GR in mitosis remain controversial [23]–[28].

Here we show tight coupling of G1 progression to GR nuclear translocation, with rapid exclusion at mitosis and into early G1. This was accompanied by loss of transactivation of endogenous target genes in mitosis-synchronised cell populations, and altered kinetics of PKB and ERK activation. There was also a striking increase in ligand-independent, selective transactivation of a concatemeric reporter gene. This was due to altered function of GR AF-1, the site of two residues known to be targets of CDKs [19], [24], [25], [29]. Mutation of one of these sites, serine 211, abolished this effect, whereas mutation of the other, serine 203, had no impact. Taken together we show strong cell-cycle phase regulation of GR function mediated, in part, by phosphorylation of GR on serine 211. This has clear implications for Gc action in rapidly dividing cells.

## Results

### GR is subject to ligand-independent, cell cycle dependent trafficking

**Figure 1:**
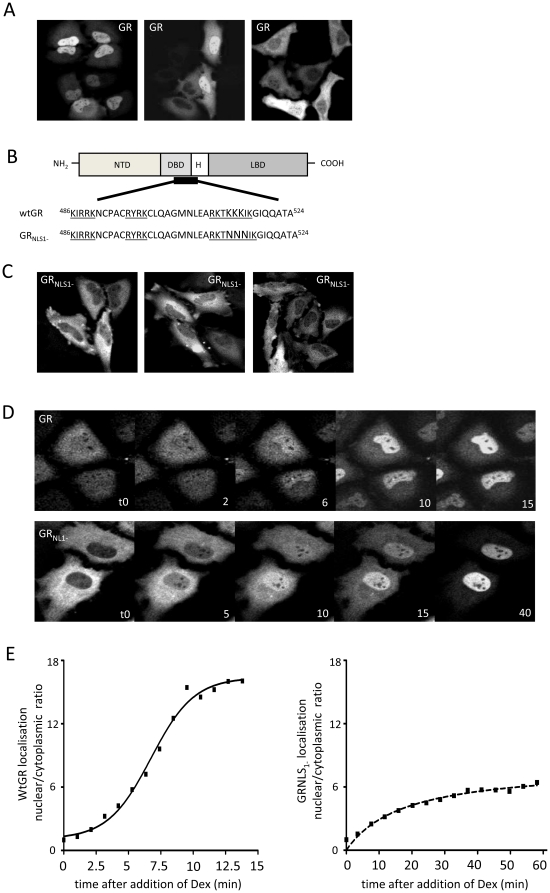
Ligand-independent Gr nuclear translocation requires NLS1. (A) HeLa cells were transfected with 1 µg EGFP-GRα and cultured in growth media containing 10% charcoal dextran stripped FCS (CSS). (B) An NLS deficient GR (GR_NLS1_-) was generated by site directed mutagenesis. NTD; N-terminal domain, DBD; DNA binding domain, H; hinge region, LBD; ligand binding domain. (C) HeLa cells were transfected with 1 µg EGFP-GR_NLS1_- and cultured in growth media containing 10% CSS. (D) HeLa cells were transfected with 1 µg EGFP-GRα or EGFP-GR_NLS1_- and cultured in growth media containing 10% CSS alone, or in the presence of 10 nM dex (time indicated in minutes). (E) Cells were analysed for GR localisation in real time and nuclear translocation of Wt GR and GR_NLS1_- quantified as a nuclear/cytoplasmic ratio. In A and C, multiple representative fields are shown.

Live cell imaging using a transfected fluorophore-tagged GR (GR-EGFP) illustrates the heterogeneity of GR distribution at rest (Fig. 1A) with some cells having strict nuclear exclusion, and others having near equal nuclear/cytoplasmic partition. Mutation of nuclear localisation signal 1 (NLS1, Fig. 1B) prevented any ligand-independent nuclear accumulation rendering GR entirely cytoplasmic in the absence of ligand (Fig. 1C). Although ligand-independent GR shuttling required NLS1, ligand-dependent nuclear translocation was only partially impaired suggesting dissociation between the two mechanisms (Fig. 1D, E).

**Figure 2:**
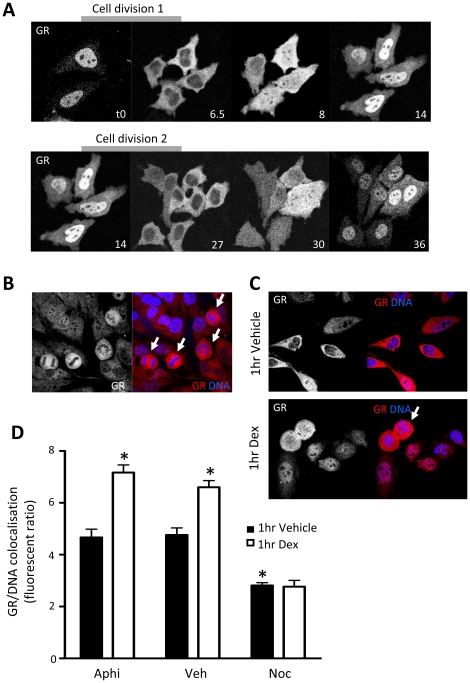
Subcellular GR localisation is synchronous with cell cycle phase. (A) HeLa cells were transfected with 1 µg EGFP-GRα and cultured in growth media containing 10% CSS. Cells were analysed for GR localisation in real time. Cells received no treatment, therefore time is indicated in hours from an arbitrary start point. (B, C) Unsynchronised HeLa cells cultured in CSS were treated with vehicle or 100 nM dex for 1 hour, then fixed and endogenous GR labelled (red). DNA was counterstained with DAPI (blue). Representative images of mitotic cells (indicated by arrows) are shown. (D) HeLa cells cultured in CSS were treated with vehicle (Veh), aphidicolin (Aphi) or nocodazole (Noc) for 15 hours, then washed and incubated with vehicle or 100 nM dex for an additional 1 hour. Cells were fixed with PFA, endogenous GR labelled using a specific antibody and DNA stained with Hoechst. Cells were subjected to high content analysis using an algorithm which analysed GR subcellular distribution against DNA distribution (nucleus in interphase cells, and condensed chromosomes in mitotic cells). Graph depicts mean +/− SD of three independent experiments performed in duplicate (>40,000 cells in each case). * indicates p<0.05 compared to vehicle control.

More detailed studies identified a dramatic and unexpected nuclear translocation that was independent of added ligand, but synchronous with cell cycle progression (Fig. 2A). The slow rate of nuclear import contrasts with the very rapid translocation seen in response to ligand binding, taking hours as compared to minutes. Peak nuclear accumulation of GR was seen immediately before cell division and was strictly cytoplasmic following cell division (supporting online material, Movie S1). Cross-sectional analysis of endogenous GR intracellular distribution in fixed cells also revealed heterogeneity in GR localisation with significant nuclear GR in some cells at rest (Fig. 2B, C). In addition, cells immediately post cell division, identified by rounded cell morphology and condensed chromosomes had exclusively cytoplasmic GR, which failed to translocate to the nucleus even in the presence of the synthetic Gc, dexamethasone (dex, Fig. 2C). High content analysis of fixed cells labelled with a GR antibody and DNA counterstained with DAPI confirms that enriching for mitotic cells by gating with nocodazole significantly reduces the proportion of cells where GR colocalises with DNA (Fig. 2D), suggesting exclusion of GR from DNA. Similarly, while treatment of normally cycling (vehicle) or Go/G1 cells (aphidicolin) with 100 nM dex for 1 hour increases the proportion of cells that have nuclear GR, cells sychronised with nocodazole appear refractory to Gc treatment (Fig. 2D). This suggests an overriding physiological regulation of endogenous GR trafficking by the cell cycle, and suggests that cell cycle phase may be an important regulator of Gc sensitivity.

### GR function is altered in mitosis

**Figure 3:**
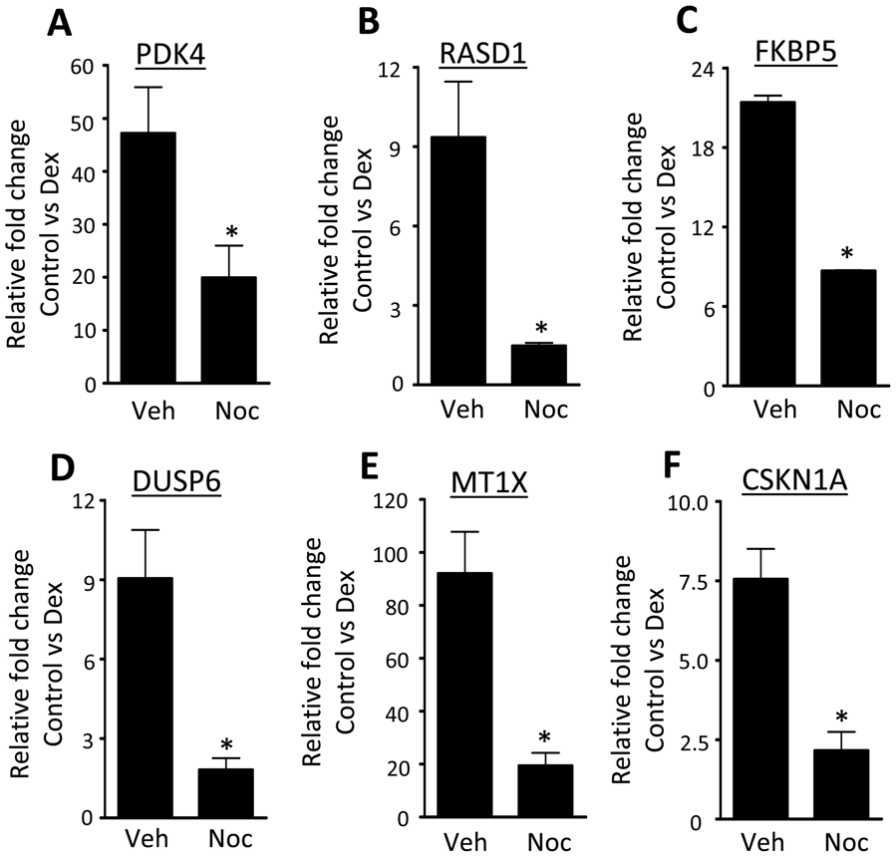
GR transactivation is dependent on cell cycle phase. HeLa cells were treated with nocodazole for 16 hours, washed and released into mitosis in the presence of vehicle or 100 nM dex for 4 hours. Cells were lysed, RNA extracted and analysed by qPCR using primers specific to PDK4 (A), RasD1 (B), FKBP5 (C), Dusp6 (D), MT1X (E) and CSKN1A (F). Graphs depict mean +/− SEM and are representative of three independent experiments. * indicates p<0.05 compared to vehicle control.

GR localisation is an important determinant in the cellular response to Gc, and so the effect of cell cycle on GR action was explored. In comparison to asynchronously growing cells, dex-induction (4 hours) of the endogenous Gc target genes PDK4, RASD1, FKBP-5, DUSP-6, MT1X and CSKN1A was significantly impaired in cells gated with nocodazole, and released into mitosis (Fig. 3A–F respectively).This finding is consistent with chromatin condensation during mitosis preventing access to the ligand activated GR. However, GR also mediates non-genomic effects, which are not influenced by changes in chromatin structure.

**Figure 4:**
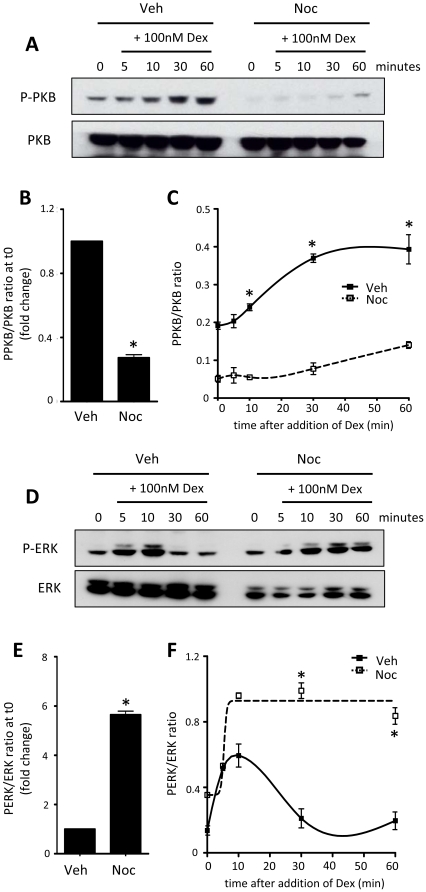
GR regulation of kinases is dependent on cell cycle phase. A549 (A–C) or HeLa (D–F) cells were treated with vehicle or nocodazole for 16 hours, washed and released into mitosis in the presence of 100 nM dex for up to 60 minutes. Cells were lysed and immunoblotted for phospho-PKB and PKB (A), or phospho-ERK and ERK (D). Immunolabelling was quantified by densitometry using ImageJ, where both cell cycle effects (B, E) and Gc-dependent effects (C, F) on kinase activity are depicted. * indicates p<0.05 compared to vehicle control.

Coupling of intracellular kinases to Gc action is cell-type specific. Treatment of A549 cells with dex induced rapid phosphorylation of PKB. In mitosis-enriched cells the basal activity of PKB was greatly reduced, as was the maximal response to dex, but there was clearly still a response, and with a similar time course in both cell populations (Fig. 4A–C). Treatment of HeLa cells with dex-induced phosphorylation of ERK. Interestingly, in cells enriched for mitosis by gating with nocodazole the kinetics of ERK activation were significantly altered, with marked prolongation of activation (Fig. 4D–F). This difference suggests a selective change in GR function dependent on cell cycle phase.

**Figure 5:**
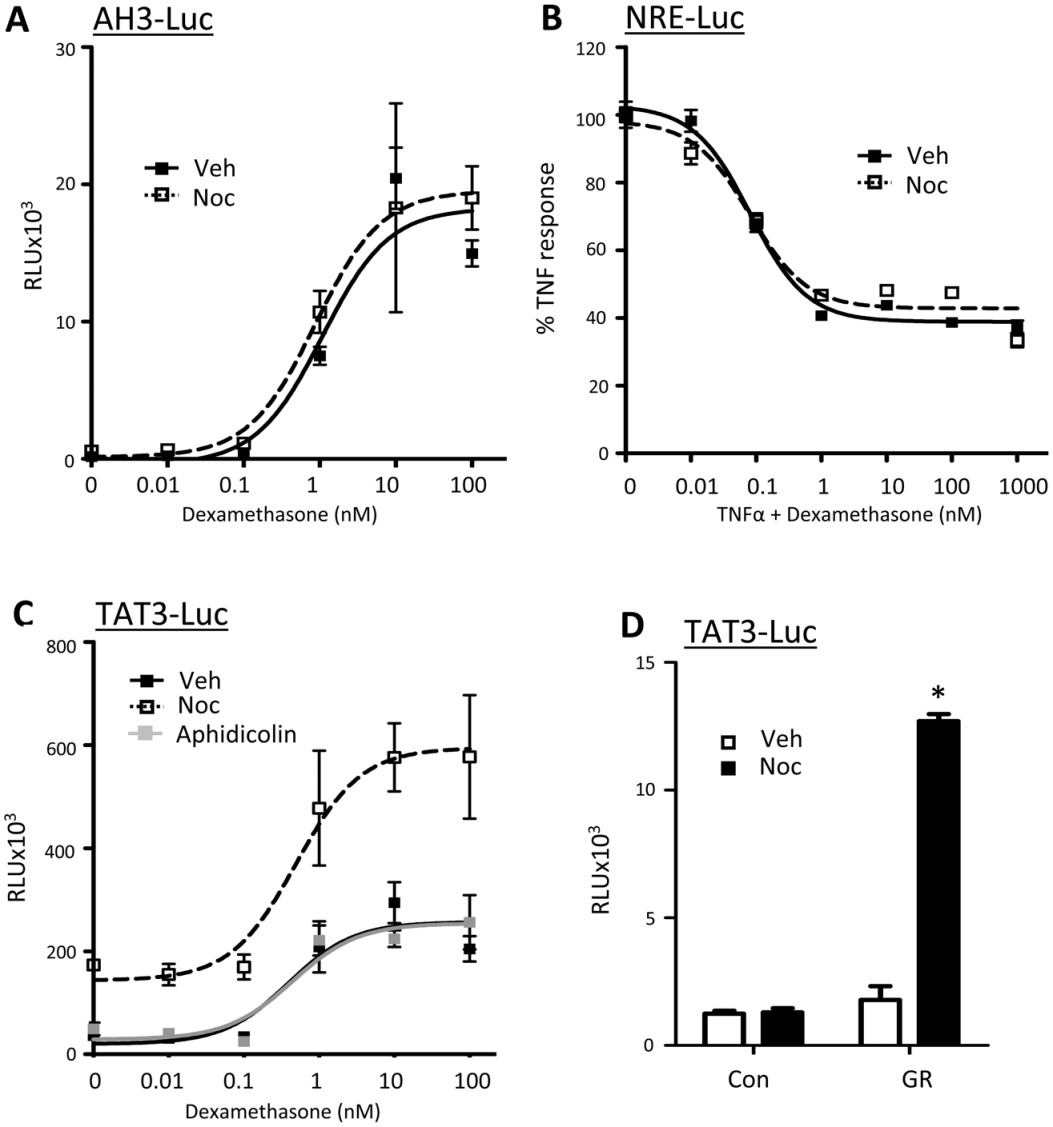
Endogenous GR activity is altered in mitosis. (A–C) HeLa cells were transfected with either 1 µg AH3-Luc (A), NRE-Luc (B) or TAT3-Luc (C) and 0.5 ug CMV-renilla (to control for transfection efficiency). Cells were treated with nocodazole or aphidicolin for 16 hours, then washed and treated with appropriate vehicle, 0.5 ng/ml TNFα (NRE-luc only) and/or dex for 16 hours. Cells were lysed, harvested and assayed for luciferase activity using a dual-luciferase reporter assay system. (D) GR deficient HEK cells were co-transfected with 1 µg TAT3-Luc and 0.5 µg CMV-renilla (to control for transfection efficiency) together with 1 µg full length GR (GR) or empty vector (Con). Cells were treated with vehicle or nocodazole for 16 hours, then washed and treated with vehicle or dex for 16 hours. Cells were lysed, harvested and assayed for luciferase activity using a dual-luciferase reporter assay system. Graphs depict mean +/− SEM and are representative of three independent triplicate experiments. * indicates p<0.05 compared to vehicle control.

A series of transient reporter gene assays were undertaken to provide a robust measure of GR activity independent of potentially non-specific effects due to chromosome condensation. MMTV (pAH3-luc) is a simple GR dimer dependent transactivation target for GR, and NFkB (NRE-luc) is a consensus NFkB target gene subject to Gc repression by a tethering mechanism requiring GR interaction with RelA [19], [23], [30]. Neither MMTV-luciferase (pAH3-luc, Fig. 5A) or NFκB-luciferase (NRE-luc, Fig. 5B) reporter genes showed any significant effect of cell cycle synchronisation on response to Gc treatment. There was however a marked and consistent induction in ligand-independent transactivation of the simple TAT3-luc reporter, which comprises three tandem repeats of a consensus GRE upstream of a minimal promoter [30], [31] (Fig. 5C). This effect was specific to G2/M synchronisation, since it was evident in cells gated at G2/M with nocodazole, and then released into mitosis, but not with aphidicolin treatment (Fig. 5C) which synchronises cells in G1. This effect was also GR specific, as ligand-dependent induction of TAT3-luc in GR deficient HEK cells required exogenous GR (Fig. 5D).

**Figure 6:**
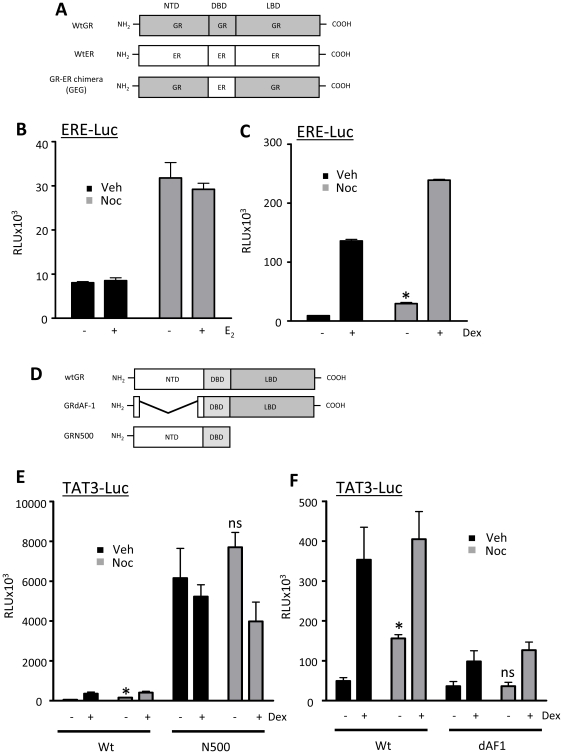
GR N-terminal is regulated by mitosis. (A) A GR-ER chimera was used which comprises the GR N- and C-terminus and the ER DNA binding domain. (B, C) HeLa cells were co-transfected with a receptor chimera (GEG), together with 1 µg of the estrogen receptor responsive reporter ERE-Luc and 0.5 µg CMV-renilla (to control for transfection efficiency). 24 hours later, cells were treated with vehicle or nocodazole for 16 hours, then washed and treated with vehicle, estradiol or dex for 16 hours. Cells were lysed, harvested and assayed for luciferase activity using a dual-luciferase reporter assay system. (D) Two deletion mutant receptors were used which lack either the C-terminal ligand binding domain (GRN500) or the AF-1 domain within the N-terminus (GRdAF1). To minimise confounding effects of wildtype endogenous GR in HeLa cells, GR deficient HEK cells were used in this instance. HEK cells were co-transfected with 1 µg TAT3-Luc and 0.5 µg CMV-renilla (to control for transfection efficiency) together with either 1 µg full length GR, GRN500, (E), or GRdAF1 (F). 24 hours later, cells were treated with vehicle or nocodazole for 16 hours, then washed and treated with vehicle or dex for 16 hours. Cells were lysed, harvested and assayed for luciferase activity using a dual-luciferase reporter assay system. Graphs depict mean +/− SEM and are representative of three independent triplicate experiments. * indicates p<0.05 compared to vehicle control.

To explore the role of the GR DNA binding domain (DBD) a chimeric GR was used, where the GR DBD was substituted for the corresponding DBD of the estrogen receptor (ER, Fig. 6A). Although wildtype ER transactivates ERE-luc in response to estradiol, the GR-ER chimera (GEG) fails to respond to estradiol (Fig. 6B) and instead binds, and transactivates ERE-luc in response to dex. These studies revealed that the ligand-independent activity of GR with G2 synchronisation was still evident following substitution of the GR DBD with ER DBD (Fig. 6B, C). This suggested that the mitosis specific GR activation was mediated through either the C terminal LBD or the GR N terminal which contains the major transactivation domain, AF-1. Deletion constructs for both GR C and N terminal domains were used (Fig. 6D). These showed that loss of the C terminal resulted in a constitutively active transactivator on which no cell cycle effect was observed (Fig. 6E). Loss of GR AF-1 also abolished the ligand-independent transactivation of the TAT3-luc reporter (Fig. 6F). This implicates the GR AF-1 as the target for cell cycle regulation of GR function.

### S211GR is required for cell cycle dependent, ligand-independent activity

**Figure 7:**
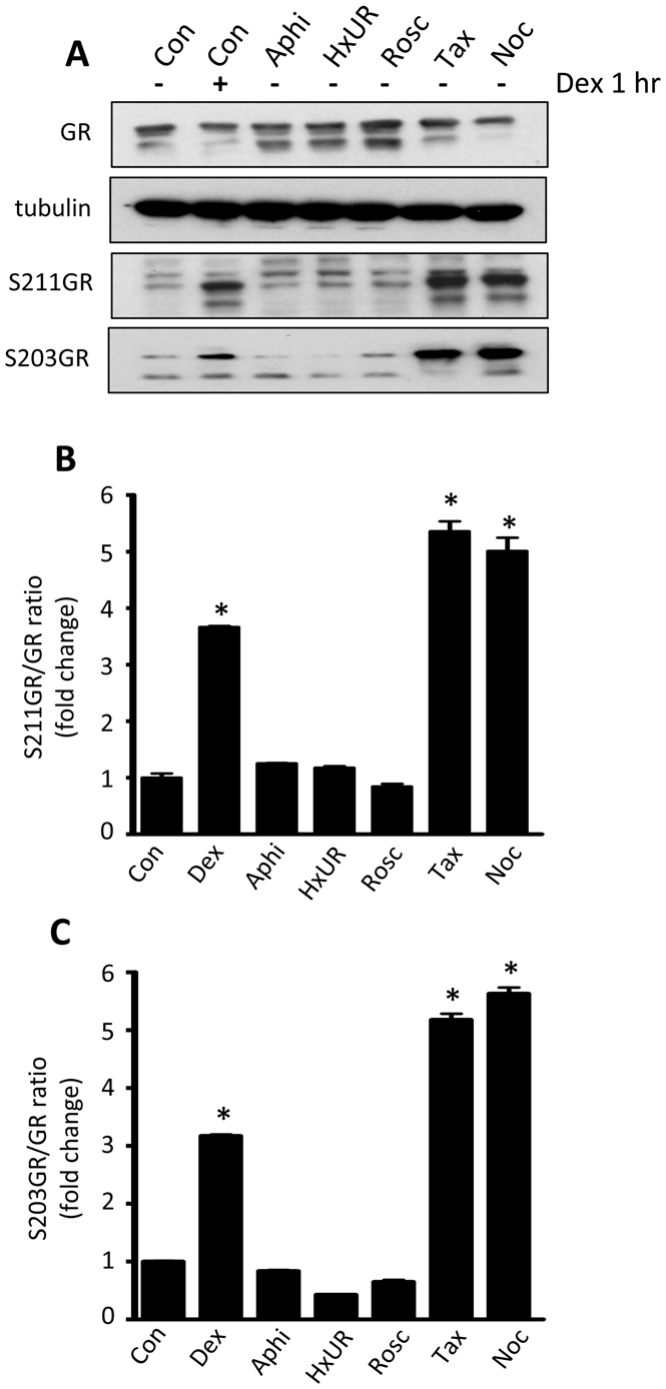
Synchronisation in mitosis induces GR phosphorylation. (A) HeLa cells were treated for 16 hours with either vehicle (Veh), aphidicolin (Aphi), hydroxyurea (HxUR), roscovitine (Rosc), taxol (Tax) or nocodazole (Noc) then lysed and immunblotted for GR, phospho-S203GR (S203GR), phospho-S211GR (S211GR) and tubulin. 1 hour treatment with 100 nM dex was used as a positive control. Immunoreactive bands for S211GR (B) and S203GR (C) were quantified by densitometry using ImageJ. Representative images are shown. * indicates p<0.05 compared to vehicle control.

The AF-1 domain harbours multiple phosphorylation sites. The two best-characterised phosphorylation sites in the GR AF-1 domain are serines 203 and 211, both of which are important modulators of GR-mediated transactivation [24]. Using phospho-specific antibodies we demonstrate a ligand independent induction of both phospho-S211GR (Fig. 7A, B) and phospho-S203GR (Fig. 7A, C) that is specific to mitotic cell cycle arrest (nocodazole- and taxol-gating), rather than a consequence of cell cycle arrest, since aphidicolin, hydroxyurea and roscovitine which arrest earlier in the cell cycle were without effect. Interestingly, the magnitude of either S203GR or S211GR phosphorylation following mitotic arrest was similar to that seen following 1 hour dex treatment (Fig. 7A–C).

**Figure 8:**
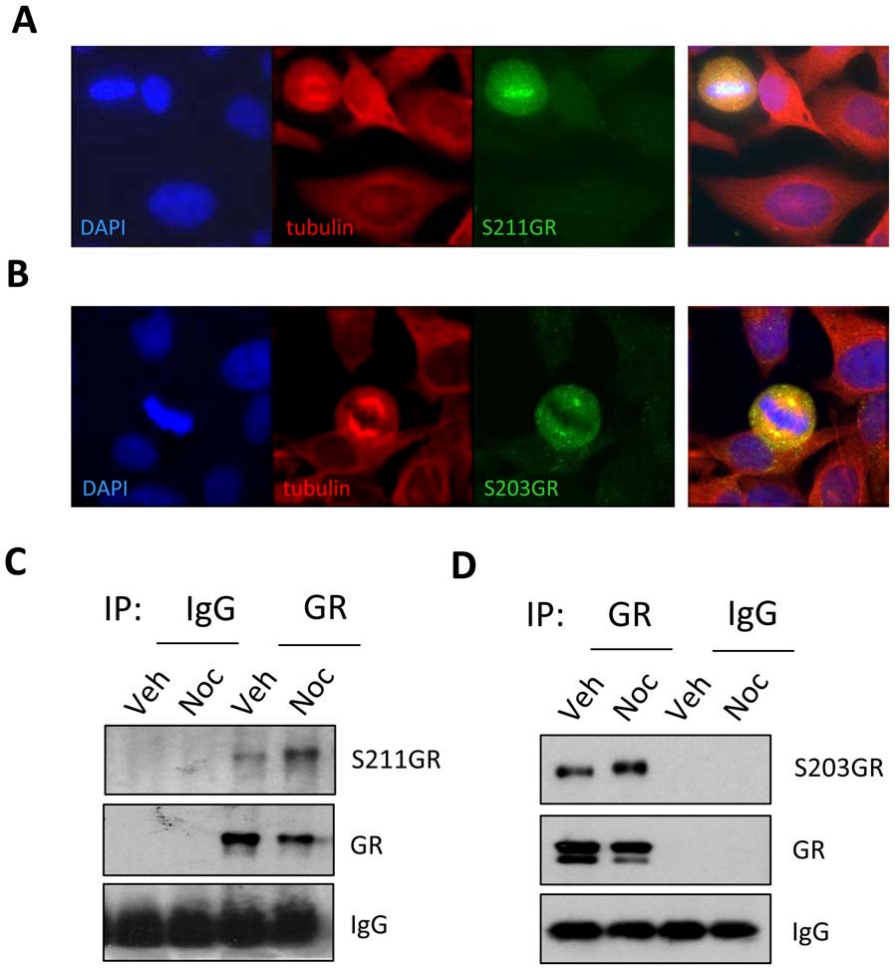
GR is phosphorylated in mitotic cells. Untreated HeLa cells cultured in CSS were double labelled with antibodies specific to tubulin and phospho-S211GR (S211GR, A) or phospho-S203GR (S203GR, B) and DNA counterstained with DAPI. Representative images of mitotic cells within a mixed population are shown. (C, D) HeLa cells were treated with vehicle (Veh) for 16 hours or synchronised with nocodazole (Noc) for 16 hours, then washed and released into mitosis. Vehicle treated cells were scraped into RIPA buffer and a pure population of mitotic cells collected from the nocodazole treated population by shake off, then pelleted and lysed in RIPA. Lysates were immunoprecipitated for GR or control IgG and precipitates electrophoresed and immunoblotted for GR together with phospho-S211GR (C) or phospho-S203GR (D). Images and immunoblots are representative of at least three independent experiments.

Examination of individual cells within a normally cycling population reveals a striking and very specific induction of both S211GR (Fig. 8A) and S203GR (Fig. 8B) phosphorylation in mitotic cells when compared to surrounding, non-dividing cells. Immunoprecipitation-immunoblotting experiments were used to confirm the specificity of both the phospho-S211GR (Fig. 8C) and S203GR (Fig. 8D) antibodies, and also show induction of GR phosphorylation in mitosis-enriched cell populations (Fig. 8C, D).

**Figure 9:**
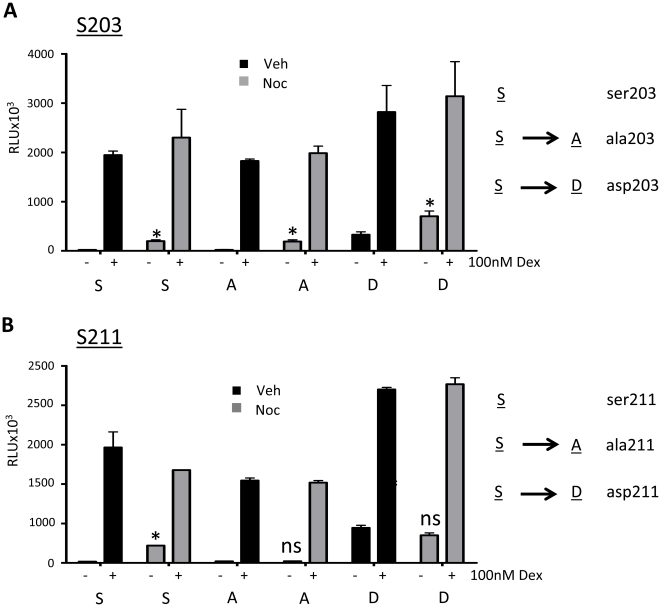
Mitosis driven phosphorylation modifies GR activity. To minimise confounding effects of wildtype endogenous GR in HeLa cells, GR deficient HEK cells were used in this instance. (A, B) HEK cells were co-transfected with 1 µg TAT3-Luc and 0.5 µg CMV-renilla (to control for transfection efficiency) together with either wildtype GR (S203GR, S211GR), a phosphorylation deficient receptor (A203GR, A211GR) or a phosphorylation mimic (D203GR, D211GR). 24 hours later, cells were treated with vehicle or nocodazole for 16 hours, then washed and treated with vehicle or dex for 16 hours. Cells were lysed, harvested and assayed for luciferase activity using a dual-luciferase reporter assay system. Graphs depict mean +/− SEM and are representative of three independent triplicate experiments. * indicates p<0.05 compared to vehicle control.

To determine if either of these sites were responsible for the ligand-independent activity of GR on TAT3-luc, point mutations of these two phosphorylation sites were generated, substituting serine for either alanine (S to A) or aspartate (S to D) to generate phosphodeficient or phosphomimetic mutants respectively. The point mutated GR expression vectors were co-transfected with TAT3-luc into cells deficient in endogenous GR, HEK293 cells. Mutation of serine 203 to alaninine (A203GR) or aspartate (D203GR) had no effect on the induction of TAT3-luc transactivation observed in mitosis-enriched cells (Fig. 9A). In contrast mutation of serine 211 completely abolished the effect of mitosis. A211GR failed to induce the mitosis dependent increase in reporter gene activity and D211GR promoted ligand independent GR transactivation, where no further induction was seen in nocodazole gated cells (Fig. 9B). Neither phosphodeficient A211GR, nor phosphomimetic, D211GR impaired Gc mediated transactivation of the reporter gene. These data suggest that induction of phospho-S211GR in mitosis drives ligand-independent GR activation.

**Figure 10:**
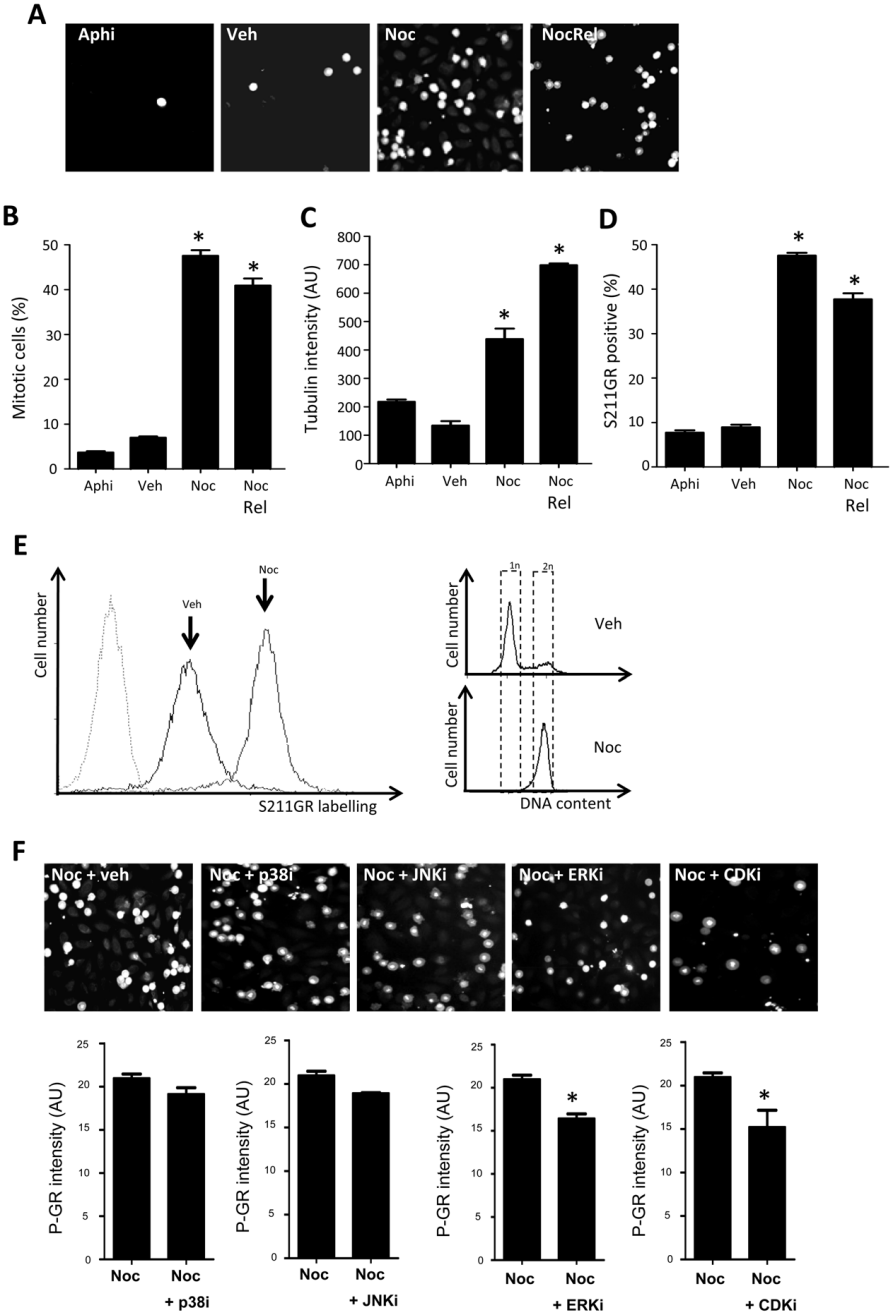
Induction of GR phosphorylation directly correlates with mitotic index. HeLa cells cultured in CSS were treated with vehicle (Veh), aphidicolin (Aphi), nocodazole for 16 hours (Noc) or nocodazole for 15 hours followed by vehicle for 1 hour (Noc Rel) and fixed with PFA. Cells were double labelled with antibodies specific to tubulin and phospho-S211GR, and DNA counterstained with Hoechst. Representative images of S211GR are shown (A). Cells were gated according to DNA content, and the percentage of mitotic cells (B), average tubulin intensity (C) and induction of phospho-S211GR (D) measured using a high content algorithm. Graphs depict mean +/− SEM of three independent triplicate experiments (>100,000 cells). (E) HeLa cells were treated with vehicle (Veh) for 16 hours or synchronised with nocodazole (Noc) and released into mitosis. Asynchronously dividing vehicle cells were collected following trypsinisation and nocodazole treated mitotic cells were collected by shake off. Samples were divided into two, and either fixed with ethanol or PFA, followed by methanol. Ethanol fixed samples were stained with PI then analysed by FACS for DNA content and PFA/Methanol fixed cells were labelled with a phospho-S211GR specific antibody or rabbit IgG and analysed by FACS for phospho-S211GR staining. (F) HeLa cells cultured in CSS were treated with nocodazole (Noc) for 15 hours followed by vehicle for 1 hour together with 10 µM kinase inhibitor (as indicated) and fixed with PFA. Cells were double labelled with antibodies specific to tubulin and phospho-S211GR, and DNA counterstained with Hoechst. Representative images of S211GR are shown. Cells were gated according to DNA content and phospho-S211GR measured using a high content algorithm. Graphs depict mean +/− SEM of three independent triplicate experiments (>100,000 cells). * indicates p<0.05 compared to vehicle control.

These data predict that enrichment of cells in mitosis will result in a marked increase in the frequency of phospho-S211GR positive cells, and that the phospho-211GR positive cells will correlate with mitotic cell numbers. To test this we gated cells using nocodazole (Fig. 10A, B), and either performed immediate analysis, or released the cells from nocodazole block in order to enable them to complete mitosis. The cells released showed a decrease in mitotic cells (Fig. 10B) suggesting progression through mitosis, an induction of tubulin (Fig. 10C) indicating reversal of tubule poison effects, and a concomitant decrease in phospho-S211GR positive cells (Fig. 10D). This was supported by FACS analysis (Fig. 10E).

Mitotic gating with nocodazole followed by release into either vehicle or a range of kinase inhibitors for 1 hour prior to fixation implicates CDK and ERK MAP kinases, but not p38 or JNK MAPK in driving the increase in S211GR phosphorylation (Fig. 10F).

**Figure 11:**
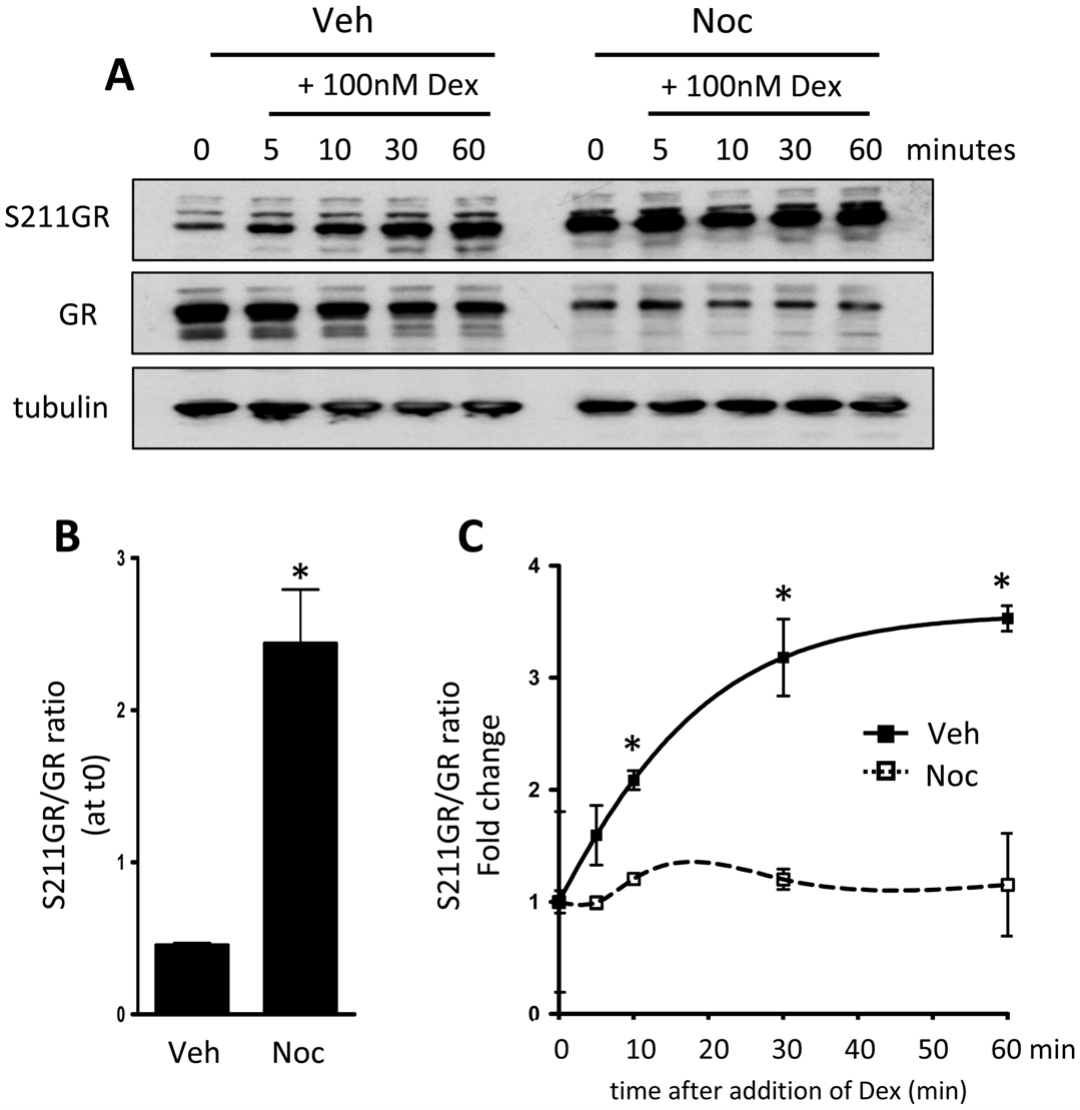
Ligand-independent GR activation in mitosis impairs activation in response to Gc. HeLa cells were treated with vehicle or nocodazole for 16 hours, then washed and released into mitosis in the presence of 100 nM dex for up to 60 minutes. Cells were lysed and immunoblotted for GR or phospho-S211GR (A). Tubulin was included as a loading control. Immunoreactive bands were quantified by densitometry using ImageJ, where both cell cycle effects (B) and Gc-dependent effects (C) on S211GR activity are depicted. * indicates p<0.05 compared to vehicle control.

Given the significant increase in S211GR phosphorylation in mitotic cells, a possibility is that in cells with mitosis-driven phosphorylation of GR at Ser211 there would be no further augmentation of GR phosphorylation seen in response to ligand binding. Accordingly, cells were gated with nocodazole, and released with or without Gc treatment. Nocodazole caused the predicted induction of phospho-S211GR, and there was no further increase seen with Gc incubation, in contrast to the control interphase cells which showed low basal phosphorylation, and rapid induction following Gc exposure (Fig. 11). Cell cycle phase is therefore an important regulator of GR activity, in both the liganded and unliganded state.

Taken together we define cell cycle driven subcellular GR trafficking and post-translational modification which in turn drive specific changes in GR function that alters the cellular response to Gc.

## Discussion

This study aimed to define the mechanism underlying nuclear trafficking of the ligand free GR, and the consequences of such trafficking for the cell. We now report direct coupling of cell cycle progression to GR subcellular localisation, phosphorylation and function.

Real time imaging studies in normally cycling cells demonstrated NLS1-dependent, ligand-independent shuttling of GR between the nucleus and cytoplasm where GR was nuclear in interphase cells, and strictly cytoplasmic immediately following completion of mitosis. Analysis of cells during mitosis showed exclusion of bulk GR from condensed chromosomes. These observations were made in cells expressing an GFP tagged GR, but compatible findings were present in untransfected cells, either grown asynchronously, or in the presence of cell cycle synchronisation agents. By using these various approaches we were able to exclude the impact of GR expression levels, fixation method, and also the potential effects of spindle toxins on the distribution and trafficking of the GR.

In these studies cells immediately post mitosis showed cytoplasmic GR, even in the presence of Gc. This observation conflicts with the accepted view that GR remains cytoplasmic until activated by ligand. However, the kinetics of rapid export at the onset of mitosis, slow import through G1 and the requirement of NLS1 for these ligand-independent effects all suggest a mechanism distinct from ligand-induced GR trafficking.

In interphase cells the movement of GR from the cytoplasm to the nucleus in response to ligand binding follows rapid kinetics, with complete translocation within minutes [19], [15]. This mechanism requires binding of importin α, and association with the nuclear pore complex. More recent studies have suggested constitutive trafficking of non-ligand bound GR, in association with heat shock proteins, through the nuclear pore, with the steady state distribution determined by fine tuning of import and export rates [15], [20]. Ligand bound GR remains nuclear for hours even after ligand withdrawal, a phenomenon now attributed to a specific nuclear retention signal close to but separable from the NLS1 in the hinge region [21]. Therefore, the paradoxical kinetics of movement observed in our studies is striking, with slow kinetics of nuclear import through G1, and very rapid expulsion of the GR from the nucleus immediately before mitosis, with persisting strict exclusion in early G1 in the resulting daughter cells.

There have been a number of previous reports relating cell cycle phase to GR function, including hormone binding, nuclear translocation, post-translational modification, and transcriptional regulatory activity [27], [28], [32]–[36]. Previously the effect of cell cycle phase has principally focussed on the transcriptional regulatory activity of the GR. There have been some inconsistencies in the literature, as various cell synchronisation protocols have been used which may have other toxic effects on target cells [28]. This has remained controversial, with studies suggesting that mitotic repression of GR transcriptional activity is a non-specific consequence of chromatin condensation [28], and that GR would function correctly if chromatin were more readily accessible.

Cell synchronisation with nocodazole, followed by release into mitosis with or without Gc clearly demonstrated impaired induction of six Gc regulated index genes, when compared to asynchronous cell populations. These results are certainly compatible with some of the earliest observations of impaired Gc action in mitosis [37], [38], but mechanistic insight is limited due to the effects of chromosome condensation.

Therefore, we have also defined rapid non-transcriptional GR effects which would not be affected by chromosome condensation. HeLa cells show non-genomic coupling of Gc to ERK phosphorylation, whereas in A549 cells there is coupling to PI3kinase [23]. Nocodazole synchronisation altered the kinetics of activation of MAP kinase in HeLa cells, and exerted a global inhibition of PKB phosphorylation in A549 cells so that while there was still an induction in PKB phosphorylation following Gc treatment, the magnitude of response was reduced. This implied a specific alteration in GR-kinase coupling in mitosis enriched cell populations, suggestive of a change in function rather than an effect mediated by steric hindrance of GR binding to target sites in the genome. Whilst promising, a semi-quantitative technique is not suitable to map potentially small changes in GR activity, and so a more sensitive model was sought.

GR is a ligand activated transcription factor, and as such transcriptional effects of Gc are the best characterised. A transient reporter gene system was selected as the construct was not chromatinised, and so not subject to condensation. We chose to use three well-characterised, simple reporter genes. MMTV and TAT3 are both targets for GR transactivation, but show differential response to targeted mutations that disrupt dimerisation [31], [39], [40]. In addition, we chose an NFkB reporter gene, as this is repressed by monomeric GR binding to the RelA component of NFkB [30].

We observed no cell cycle effect on transactivation of an MMTV reporter gene or transrepression of an NFkB reporter. However, there was a striking induction of ligand independent promoter activity of a simple reporter consisting of three repeats of the tyrosine aminotransferase GRE; TAT3-Luc.

This was particularly interesting as a gain of function supports the theory of a specific, regulated, potentially reversible change to GR that would modify the cellular response to Gc. To define the underlying mechanism, a series of GR deletants, and chimeras were used. These revealed a cell cycle driven transactivation function localised to the GR N-terminal AF-1 domain. The AF-1 domain contains the major transactivation function of the GR, and is known to be a site of post-translational modification.

We went on to show that within the GR AF-1 domain two residues, serine 203 and serine 211, are phosphorylated both in response to ligand activation, and following entry into mitosis. We were able to show induction of phospho-S203GR and -S211GR by immunoblot analysis after gating with nocodazole, and also in naturally cycling populations of cells by immunofluorescence. FACS and high content cell imaging demonstrated that all mitotic cells underwent ligand-independent S211GR phosphorylation, suggesting no additional factors were required.

The cell cycle phase regulation of S211GR phosphorylation was dominant over ligand induced changes, as shown by comparing a time course of dex induction of phospho-S211GR in the presence and absence of nocodazole synchronisation.

There is evidence from the literature that CDK, and p38 MAP kinases can phosphorylate GR on S211 [29], [41]. During mitosis CDKs are activated, and are therefore highly likely to be playing an important role. Although inhibition of p38 had no effect, we show that inhibition of either CDK, or ERK, impairs S211 phosphorylation in mitosis. A more comprehensive analysis was not possible as complete ablation of CDK or ERK activity also impacted cell cycle progression.

Closer examination of cells in mitosis revealed differential distribution of phospho S203GR, and phospho S211GR, with phospho S203GR localised to the spindle poles, and phospho S211GR aligning along the condensed chromosomes. This differential distribution of the two phosphoforms has not been observed before in mitosis. In interphase cells however, the two phospho marks appear to identify different sub-populations of GR [42], perhaps suggesting a common targeting mechanism for GR in mitotic and interphase cells.

There was a notable decrease in total GR immunoreactivity in nocodazole gated cells. This was a consistent and robust finding. This would provide a very simple explanation for a loss of GR function. However in mitosis we did not observe a global reduction in Gc actions but show very selective effects, in particular on GR-kinase coupling and transactivation of TAT3-luc. This is not compatible with downregulation of GR. It does raise the possibility however that phosphorylation of GR in mitosis couples the GR to ubiquitination, and degradation, in a similar manner to that described for the ligand bound, and activated GR.

We predicted that the cell-cycle dependent, ligand-independent transactivation may be mediated through phosphorylation of one or both of these residues, and the subsequent recruitment of co-modulator proteins such as MED14 [24], [43]. The mediating role of S211GR and S203GR phosphorylation events were defined using site directed mutagenesis in GR deficient HEK293 cells. These studies revealed that S203GR played no role, but in contrast S211GR to A211GR substitution completely abolished the ligand independent induction of promoter activity, and the phosphoS mimetic D211GR induced promoter activity above that seen with wild-type GR, where no further induction was seen in mitosis. S211GR phosphorylation therefore selectively mediates the cell cycle dependent, ligand-independent induction of transactivation. It is perhaps not surprising that induction of phospho-S211GR was important for transactivation, as it is already known that this modification is required for GR binding to certain DNA targets [42], [43].

Demonstration of ligand-independent transcriptional regulation by the GR has also been recently described in epithelial cells activated by TNFalpha. The mechanism described in this report requires phosphorylation of S226, and not S211, and no role for cell cycle is proposed [44]. Therefore, our findings are entirely novel, but certainly not incompatible.

Regulation of cell cycle progression and cell fate [19] is mediated by direct transcriptional regulation of p27KIP and cyclin D expression by GR and is one of the conserved actions of Gc. We now show that additionally the cell cycle feeds back to regulate GR modification, cellular trafficking and function, independent of externally derived ligand. This implies a cyclic, reversible change to GR which alters the cellular response to Gc and correlates with progression through the cell cycle. Our data indicates that maximal Gc effects are likely to occur in interphase cells, which also suggest a potential feed-forward circuit where G0/G1 cell cycle arrest mediated by Gc augments the cellular response to subsequent Gc exposure.

Here we show that cell cycle phase exerts a regulatory activity on the function of the GR. The physiological consequences of cell cycle driven GR trafficking, modification, and ligand independent activation are likely to be important. Clearly, many cumulative factors play a role in determining Gc sensitivity. The tight coupling of mitotic index, S211GR phosphorylation and GR activity may represent a very basic level regulation. Further dissecting how reversible modification of GR impacts Gc sensitivity, and delineating additional regulatory mechanisms will be invaluable to understanding the mechanisms that regulate Gc responsiveness of tissues.

## Materials and Methods

Anti-GR (clone 41) from BD Biosciences (Oxford, UK); anti-S203GR from AbCam (UK); anti-S211GR, anti-PKB, anti-PPKB, anti-ERK and anti-PERK from Cell Signalling Technology (MA, USA); anti-αtubulin from Sigma (Poole, UK); horseradish peroxidase conjugated anti-mouse and anti-rabbit from GE Healthcare (Buckinghamshire, UK); Alexa 546 conjugated anti-mouse and Alexa 488 conjugated anti-rabbit from Invitrogen (Paisley, UK). Dexamethasone and estradiol from Sigma (Dorset, UK). Nocodazole, aphidicolin, HxUR, SB202190 (p38i), JNK inhibitor VIII (JNKi), PD98059 (MEKi) and roscovitine (CDKi) from Calbiochem (UK). TAT3-Luc, AH3, ERE-Luc, EEE, GEG, GGG, and pEGFP-GRα have been described previously [19], [40]. Primer sequences available on request.

### Cell line generation, culture and maintenance

Human cervical carcinoma cells (HeLa; ECACC, Wiltshire, UK), lung epithelial cells (A549, ECACC) and embryonic kidney cells (HEK, ECACC) were cultured in Dulbecco's modified Eagle's medium (DMEM) containing Glutamax supplemented with 10% charcoal dextran stripped fetal calf serum (CSS, Invitrogen). Cells were maintained in a humidified atmosphere of 5% CO_2_ at 37°C.

### Cell cycle arrest

Cells were enriched for G2/M checkpoint using 16 hour treatment with the microtubule disrupting drug nocodazole, or the microtubule stabilising drug Taxol. Following accumulation of cells at G2/M checkpoint, they were carefully washed with serum free media, then returned to full culture media containing 10%CSS then treated as required. Following this, mitotic spindles reformed, and cells progressed into mitosis. All treatments were therefore conducted in cells with an intact microtubule network. Cells were also growth arrested using Hydroxyurea, Roscovitine, and Aphidicolin treatment for 16 hours. They were also carefully washed with serum free media, then returned to full culture media containing 10%CSS before treatment.

### Site directed mutagenesis

GR constructs with point mutations at S203 (A203GR, D203GR) and S211 (A211GR, D211GR) and the NLS deletant (GRNLS-) were generated using a quick change II site directed mutagenesis kit (Stratagene) using manufacturers instructions.

### Real-time fluorescent cell imaging

10^5^ HeLa cells were transfected with EGFP-GRα (1 µg) using Fugene 6 reagent (3∶1 v/w ratio). 24 hours post transfection cells were trypsinised and seeded at a density of 10^5^ cells per 35 mm glass-bottomed plate (Iwaki, Japan). Cells were imaged 24 hours after transfection on a Zeiss LSM510 Axiovert 200 M equipped with an XL incubator (maintained at 37°C, 5% CO_2_, in humid conditions) through a 63× objective (numerical aperture, 1.4; Zeiss). Excitation of EGFP was performed using an argon ion laser at 488 nm. Emitted light was captured through a 505–550 nm bandpass filter from a 540 nm dichroic mirror. Images were taken at regular intervals and data captured and analyzed using LSM510 software (Zeiss).

### Immunofluorescence

Cells were treated as specified in the results, fixed with 4% paraformaldehyde (PFA) for 30 minutes at 4°C, then permeabilised (0.02% Triton X-100 in PBS) for 30 minutes at room temperature (RT). Fixed cells were blocked (1% FCS in PBS) for 4 hours at RT with agitation, then in primary antibody (diluted in blocking buffer) overnight at 4°C. After three 10 minute washes in PBS cells were incubated in secondary antibody (diluted in PBS) for 2 hours. After incubation with Hoechst for 10 minutes, coverslips were washed three times and mounted using Vectashield aqueous hard set mountant (Vector Laboratories, Peterborough, UK). Images were acquired on a Delta Vision RT (Applied Precision) restoration microscope using a 60×/1.42 Plan Apo objective and the Sedat filter set (Chroma 89000). The images were collected using a Coolsnap HQ (Photometrics) camera with a Z optical spacing of 0.5 µm. Raw images were then deconvolved using the Softworx software and maximum intensity projections of these deconvolved images processed using ImageJ. The fluorescent intensities of nucleus and cytoplasm were compared, and the ratios presented.

### High content analysis

High Content Analysis was performed in the laboratories of Imagen Biotech (Manchester, UK) on an Arrayscan II (Cellomics, Thermofisher, USA). Cells were plated into glass bottomed 96-well plates and processed as outlined in ‘Immunofluorescence’. The percentage of mitotic cells was calculated based on a threshold of average nuclear intensity and the arrayscan compartmental analysis algorithm (Cellomics) used to determine the intensity of endogenous GR and phospho-S211GR staining. Measurements of GR subcellular distribution in mitotic and non-mitotic cells were established using appropriate nuclear gating.

### Immunoprecipitation

Whole cell extracts (500 µg protein) were pre-cleared with protein A/G-coated sepharose beads. In the test samples, supernatant was incubated with 5 µg primary antibody and protein A/G-coated sepharose beads overnight at 4°C. The control sample supernatants were incubated with protein A/G-coated sepharose beads and IgG from non immunised animals. Following incubation, protein A/G-coated sepharose beads were collected by centrifugation (1800 g) and washed three times (5 minutes) with ice-cold PBS. Samples were boiled for 5 minutes in reducing loading buffer, and the beads removed prior to electrophoresis.

### Immunoblot analysis

Cell lysates or immunoprecipitates were electrophoresed on 4–12% Tris-Glycine gels (Invitrogen) and transferred to 0.2 micron nitrocellulose membranes (BioRad Laboratories, Hertfordshire, UK) overnight at 4°C. Membranes were blocked for 6 hours (0.15 M NaCl, 1% dried milk, 0.1% Tween 20) and incubated with primary antibodies (diluted in blocking buffer) overnight at 4°C. After three 10 minute washes (88 mM Tris pH 7.8, 0.25% dried milk, 0.1% Tween 20), membranes were incubated with a species-specific horseradish peroxidase-conjugated secondary antibody (diluted in wash buffer) for 1 hour at RT, and washed a further three times, each for 10 minutes. Immunoreactive proteins were visualised using enhanced chemiluminescence (ECL Advance, GE Healthcare).

### FACS analysis

For DNA quantification, cells were trypsinised and the cell suspension combined with equal volumes of 100% ice-cold ethanol for 1 hour at 4°C. Cells were pelleted at 1500 g for 10 minutes and resuspended in 200 µl PBS. 50 µl DNAse free RNAse A (1 mM) was added to each sample and incubated at RT for 30 minutes. 50 µl propidium iodide (1 mM) was added prior to analysis. For antibody labelling, cells were trypsinised then fixed with 4% PFA for 1 hour at 4°C. Cells were post-fixed in ice-cold 50% methanol (1 hour), washed three times with PBS then blocked for 1 hour (5% FCS, 0.001% Triton-X-100, PBS). Cells were incubated in primary antibody for 1 hour (diluted in block) washed three times with PBS then incubated with secondary antibody for 30 minutes (diluted in block). Cells were washed three times in PBS prior to analysis (Beckman Coulter Cyan ADP, 488 nm excitation; 530/40 nm bandpass, 635 nm excitation; 665/20 nm bandpass). Histograms were processed using Summit v4.3 software.

### Reporter gene assay

Cells were co-transfected with 1 µg ligand-regulated Tat3-, AH3-, NRE- or ERE- firefly luciferase reporter gene construct together with 0.1 µg CMV-renilla luciferase (a consitutively expressed enzyme that controls for transfection efficiency) using Fugene 6. 24 hours later cells were treated as specified in results prior to lysis, then assayed for luciferase activity using a dual-luciferase reporter assay system following manufacturers instructions (Promega, Southampton, UK), and as previously described [45].

### q-RTPCR

HeLa cells were treated as specified in results, then lysed and RNA extracted using an RNeasy kit (Qiagen). RNA quality was established using an Agilent bioanalyser. 10 ng RNA was reverse transcribed, and subjected to qPCR using Sybr Green detection in an ABI q-PCR machine and data analysed by δδCT method as previously described [30].

## Supporting Information

Movie S1 **Real time imaging depicted in** **figure 2A** **.** HeLa cells were transfected with 1 µg EGFP-GRα and cultured in growth media containing 10% CSS. Cells received no treatment and were analysed for GR localisation in real time. GR trafficking is under cell cycle control and undergoes repeated cycles of shuttling between the cytoplasm and nucleus.
(WMV)
